# Towards an ICF core set for ADHD: a worldwide expert survey on ability and disability

**DOI:** 10.1007/s00787-015-0778-1

**Published:** 2015-10-01

**Authors:** Elles de Schipper, Soheil Mahdi, David Coghill, Petrus J. de Vries, Susan Shur-Fen Gau, Mats Granlund, Martin Holtmann, Sunil Karande, Florence Levy, Omar Almodayfer, Luis Rohde, Rosemary Tannock, Sven Bölte

**Affiliations:** Paediatric Neuropsychiatry Unit, Department of Women’s and Children’s Health, Center of Neurodevelopmental Disorders (KIND), Karolinska Institutet, Stockholm, Sweden; Division of Neuroscience, Medical Research Institute, University of Dundee, Ninewells Hospital and Medical School, Dundee, UK; Division of Child and Adolescent Psychiatry, University of Cape Town, Cape Town, South Africa; Department of Psychiatry, National Taiwan University Hospital and College of Medicine, Taipei, Taiwan; CHILD, SIDR, Jönköping University, Jönköping, Sweden; LWL-University Hospital for Child and Adolescent Psychiatry, Psychotherapy and Psychosomatics of the Ruhr University Bochum, Hamm, Germany; Learning Disability Clinic, Department of Paediatrics, Seth G.S. Medical College and K.E.M. Hospital, Mumbai, India; School of Psychiatry, Prince of Wales Hospital and University of New South Wales, Sydney, Australia; Psychiatry Section, King Abdulaziz Medical City, College of Medicine, Riyadh, Saudi Arabia; Child Psychiatric Division, Departmant of Psychiatry, Hospital de Clinicas de Porto Alegre, Federal University of Rio Grande do Sul, Porto Alegre, Brazil; National Institute of Developmental Psychiatry for Children and Adolescents, São Paulo, Brazil; Neurosciences and Mental Health Research Program, The Hospital for Sick Children, University of Toronto, Toronto, Canada; Child and Adolescent Psychiatry Stockholm, Center for Psychiatry Research, Stockholm County Council, Stockholm, Sweden

**Keywords:** ADHD, Neurodevelopmental disorder, Gender differences, Neurodiversity, Quality of life, Assessment, Psychiatry, ICD, DSM

## Abstract

This is the second in a series of four empirical studies designed to develop International Classification of Functioning, Disability and Health (ICF and Children and Youth version, ICF-CY) core sets for attention deficit hyperactivity disorder (ADHD). The objective of this stage was to gather the opinions from international experts on which ability and disability concepts were considered relevant to functioning in ADHD. An email-based survey was carried out amongst international experts in ADHD. Relevant functional ability and disability concepts were extracted from their responses and linked to the ICF/-CY categories by two independent researchers using a standardised linking procedure. 174 experts from 11 different disciplines and 45 different countries completed the survey. Meaningful concepts identified in their responses were linked to 185 ICF/-CY categories. Of these, 83 categories were identified by at least 5 % of the experts and considered the most relevant to ADHD: 30 of these were related to Body functions (most identified: attention functions, 85 %), 30 to Activities and Participation (most identified: school education, 52 %), 20 to Environmental factors (most identified: support from immediate family, 61 %), and 3 to Body structures (most identified: structure of brain, 83 %). Experts also provided their views on particular abilities related to ADHD, naming characteristics such as high-energy levels, flexibility and resiliency. Gender differences in the expression of ADHD identified by experts pertained mainly to females showing more internalising (e.g. anxiety, low self-esteem) and less externalising behaviours (e.g. hyperactivity), leading to a risk of late- and under-diagnosis in females. Results indicate that the impact of ADHD extends beyond the core symptom domains, into all areas of life and across the lifespan. The current study in combination with three additional preparatory studies (comprehensive scoping review, focus groups, clinical study) will provide the scientific basis to define the ADHD ICF/-CY core sets for multi-purpose use in basic and applied research and every day clinical practice.

## Background

Attention deficit hyperactivity disorder (ADHD) is a neurodevelopmental condition, defined by patterns of inattention, impulsivity and hyperactivity, which affects approximately 5 % of children [1, 2] and 2.5 % of adults worldwide [3]. It is associated with neuropsychological alterations [4–10] and adverse outcomes in educational, occupational and social functioning across the lifespan. More specifically, ADHD has been found to cause academic underachievement and difficulties in scholastic skills (e.g. reading, spelling, mathematics), problems with socialisation and social relationships (e.g. with family, peers, romantic relationships), an increased risk for psychiatric comorbidity (e.g. conduct disorder, mood disorder, substance use), as well as maladaptive behaviour in various areas of life (e.g. at home, in professional and educational settings) [4, 11–14].Individuals with ADHD across the lifespan have also been found to experience lower quality of life [15, 16]. On the other hand, notwithstanding the lack of scientific support [17, 18], ADHD is also purportedly linked to specific strengths, such as creativity, hyperfocusing, high levels of energy, and flexibility. Despite clear evidence that ADHD impacts ability, these impacts are heterogeneous and can differ significantly between individuals and across development. For clinical practice and research, it would be meaningful to have a standardised nomenclature and toolkit to map the functional profiles of abilities and disabilities of those with ADHD. To date, no such framework has been developed.

The World Health Organisation (WHO) International Classification of Functioning, Disability and Health (ICF) [19] was developed to serve as a comprehensive framework for the components of functioning and disability for all health-related conditions. The ICF is designed to be used in combination with the international classification of diseases and health-related problems (ICD) [20] to create a comprehensive description of an individual’s health (using ICD) and functioning (using ICF) [21]. Whilst the ICD predominantly maps health conditions to generic categories, viewing disability as a consequence of a health condition, the ICF is based on a bio-psycho-social model of functioning, which understands disability as a result of a health condition interacting with personal and environmental factors yielding certain levels and compositions of participation and activities. The ICF provides detailed classifications of ability and disability in the areas of Body functions (i.e. physiological functions of body systems), Body structures (i.e. anatomical parts of the body), Activities (i.e. execution of tasks), Participation (i.e. involvement in life situations), and Environmental factors (i.e. physical, social, and attitudinal environment). For each of these components, aspects of functioning are described in hierarchically structured categories with up to four levels of increasing detail. At the first level are chapters, which provide a general overview of the areas of functioning that are covered by the ICF. The chapters consist of second-, third- and fourth-level categories, as can be seen in the following example of an ADHD-relevant classification from the activities and participation component:

- Level 1 chapter: d5 self-care
- Level 2 category: d570 looking after one’s health
- Level 3 category: d5702 maintaining one’s health
- Level 4 category: d57022 avoiding risks of abuse of drugs or alcohol

Personal factors are also included in the bio-psycho-social model of functioning, but are not yet classified within the ICF given the large social and cultural variance associated with them [19]. Descriptions of functional categories in the ICF create a common language that can be applied by professionals from various disciplines to facilitate effective communication in the context of the assessment and treatment of conditions and health care policy issues. To capture specific functional abilities and disabilities in developing individuals, the ICF Children and Youth version (ICF-CY) was derived from the ICF by adding and expanding on the descriptions of existing ICF categories [22].

The ICF(-CY) contains over 1600 categories (Body functions, *n* = 521; Body structures, *n* = 320; Activities and participation, *n* = 543; and Environmental factors, *n* = 270) that together provide an exhaustive classification of an individual’s functioning and as such provides a valuable system for health care and research [23, 24]. However, in its current comprehensive form, the ICF(-CY) is rather impractical for daily clinical use and research purposes, with only a selection of the categories being relevant to any particular health condition. To address this, the development of ICF Core Sets was initiated; that is, the selection of ICF(-CY) categories that are considered most relevant to individuals with a particular health condition [25–27]. The development of each Core Set follows a rigorous and systematic scientific approach that comprises an expert survey (current study), a literature review (“research perspective”), focus groups (“client and family perspective”), and a clinical study (“clinical perspective”). These ensure that the process includes a wide range of professions and other stakeholders across all of the World Health Organisation (WHO) regions. The present study is, therefore, part of a larger project that will develop standardised ICF Core Sets for ADHD. ICF Core Sets for autism spectrum disorder are also being developed as a part of this project with the results reported in separate publications [28, 29]. To develop a tool that covers functional abilities and disabilities over the life span of ADHD, ICF Core Sets are designed to be equally applicable to children, adolescents and adults. A complete description of the overall ADHD ICF Core Set development process has been published in a previous issue of this journal [30].

The objective of the current study was to capture the perspective of experts in the assessment treatment of individuals with ADHD. In an email-based survey, professionals from various disciplines around the world were asked for their opinion on which aspects of functioning are to be considered essential in the assessment of ability and disability in individuals with ADHD. Together with the other three studies mentioned above, this expert survey will provide content for an international ICF core sets consensus conference, during which a group of ADHD experts from all WHO regions will follow a formal decision-making process to arrive at a consensus on the ICF-CY categories to be included in the ICF Core Sets for ADHD.

## Methods

### Design and procedure

A worldwide expert survey was conducted via email. An internet search was performed to identify contact information for internationally known ADHD experts and for centres, clinics and university departments in all WHO regions regularly involved in the assessment and treatment of individuals with ADHD. Identified organisations were contacted via email with information about the study and a request to provide contact information of eligible experts. Contact information of experts was also provided by the project Steering Committee (see acknowledgement), a group of key opinion leaders in ADHD from all WHO regions providing guidance on the project, and by the authors’ personal professional networks. Finally, snowball sampling was applied, as all contacted experts were requested to recommend additional experts to be recruited for the survey. To be considered an “expert” for the purposes of this study, potential participants were required to (1) practice in one or more of the following professions: coach (i.e. supporting individuals in achieving goals, such as improving school grades or finding a job), counsellor, nurse, occupational therapist, physician, physiotherapist, psychologist, psychotherapist, social worker, special educator, or speech and language therapist; (2) have at least 5-year experience in the treatment of individuals with ADHD; (3) be able to read and write in English to complete the survey. Identified experts were contacted via email and received information about the study and a request to participate in the survey. Those who agreed to participate were asked to fill in a reply sheet with information about their work experience (to confirm their eligibility). Experts who were recommended by organisations or expert peers received the reply sheet and the expert survey together in one message. The expert’s eligibility was confirmed in the correspondence with the organisations and the experts who had recommended them (the inclusion criteria of the study were stated in the correspondence). Eligible experts received the survey and the reply sheet as a Word or PDF file via email to fill in and return within 1 month. If necessary, the experts received up to two reminders to return their survey response and reply sheet, and, if requested, they received extended time to respond. Data collection took place between September 2013 and October 2014.

### Expert survey

The survey was delivered in three parts. Part one contained demographic questions, e.g. age, gender, years of professional experience with ADHD, and the age group of individuals with ADHD they worked with. Part two included six items about the functioning of individuals with ADHD (Table 1). These questions were specifically formulated to ensure coverage the components of the bio-psycho-social model of the ICF(-CY), and have been applied in a similar way in previous ICF core set developments. Part two of the survey included additionally two questions. One addressed potential functional strengths related to ADHD: “In your experience with individuals with ADHD, what can be the positive sides of ADHD?” The second concerned possible gender differences in ADHD related to functioning: “In your experience with individuals with ADHD, are there any aspects of their functioning and impairment that are specific to gender?” Part three of the survey contained information regarding privacy and terms of agreement for taking part in the expert survey.

**Table 1:** Questions included in the expert survey

Survey question		ICF(-CY) component
1.	In your experience with individuals with ADHD, what are the physical (e.g. motor problems, clumsiness) and mental problems (e.g. deficits in attention) they experience?	Body functions
2.	In your experience with individuals with ADHD, which parts of the body (brain included) seem affected?	Body structures
3.	In your experience with individuals with ADHD, what are the difficulties/challenges they experience in their everyday activities and involvement in society?	Activities and participation
4.	In your experience with individuals with ADHD, what about their environment and living conditions might be hindering for them?	Environmental factors—barriers
5.	In your experience with individuals with ADHD, what about their environment and living conditions might be supportive for them?	Environmental factors—facilitators
6.	In your experience with individuals with ADHD, what personal characteristics are important in the way they handle their health condition?	Personal factors

### Identification of meaningful concepts and linking to the ICF-CY

“Meaningful concepts” are concise descriptions of specific behaviours, skills or other aspects of functioning that are to be linked to ICF-CY categories. All expert survey responses were analysed to identify the meaningful concepts contained in them. These were then linked to ICF-CY categories following the formal linking rules and procedures determined by the WHO ICF Research Branch [31, 32]. These linking rules provide guidance on how to link concepts to ICF(-CY) categories, and also on what to do where linkage is not possible. Specific codes assigned to concepts in these situations are: (1) Personal factor, if the concept is not contained in the ICF(-CY), but is clearly a Personal factor as defined in the ICF(-CY); (2) ‘Not covered’, if the concept is not contained in the ICF(-CY) and also is not a Personal factor; (3) ‘Not definable’, when the information provided in the concept is not sufficient for assigning it to a specific ICF(-CY) category; and (4) Health condition, if the concept refers to a diagnosis or health condition. Given that there are many different ways to describe the same aspect of functioning, it is possible for different meaningful concepts to be assigned to the same ICF(-CY) category.

To assure the quality and consistency of results, both the identification of meaningful concepts and the linking to ICF-CY categories were conducted by two independent researchers (ES and SM) both of whom had received extensive linking training by the ICF research branch prior to the project, and who worked in parallel. These researchers compared their linking results and consensus discussions were used to resolve disagreements. Where consensus could not be reached, a third researcher (project lead, SB) was available to make the final decision. However, this option was only rarely used because disagreements were almost all resolved by discussion. Initial agreement between the two researchers (prior to consensus in case of disagreement) was 69 % for the second-level ICF-CY categories and 74 % at the level of ICF-CY chapters. Kappa coefficients and confidence intervals for the second-level categories were 0.68 (SE = 0.005) with a confidence interval of 0.67–0.69 and at ICF-CY chapter level was *ĸ* = 0.71 (SE = 0.006) with a confidence interval of 0.70–0.72. These indicate substantial agreement.

### Data analysis

Frequency analysis was used to analyse the responses to the six ICF(-CY)-based questions of the survey. The absolute number of expert survey responses for each of the ICF-CY categories was identified, along with the corresponding percentages relative to the total number of responses received. ICF-CY categories are presented at the second level. If a concept is linked to a third- or fourth-level ICF-CY category, the corresponding second-level category is reported. Because the ICF-CY is organised hierarchically, aspects of the more specific third- and fourth-level categories are included in the less specific second-level categories. Following the ICF Core Sets development guidelines [27], a second-level ICF-CY category that was identified repeatedly in one and the same expert survey response was counted only once. Consistent with WHO and previous core set development conventions, only ICF-CY categories that were identified in at least 5 % of the expert survey responses were included in the list of candidate categories. This is done to ensure that only those categories that are most relevant to a certain condition are included. Additional frequency analysis was used to explore the possible relation between ADHD age groups (as indicated by expert’s main patient group) and identified ICF-CY categories. The two additional questions regarding specific abilities related to ADHD and possible gender differences in functioning were not analysed using frequency analysis and linking. This was owing to the fact that responses were inconsistent and heterogeneous, and it was, therefore, impossible to define meaningful concepts for linking. Instead, the answers were carefully reviewed by two independent researchers (ES and SM). Then, recurring themes or patterns of answers were summarised.

## Results

### Participating experts

**Fig. 1:**
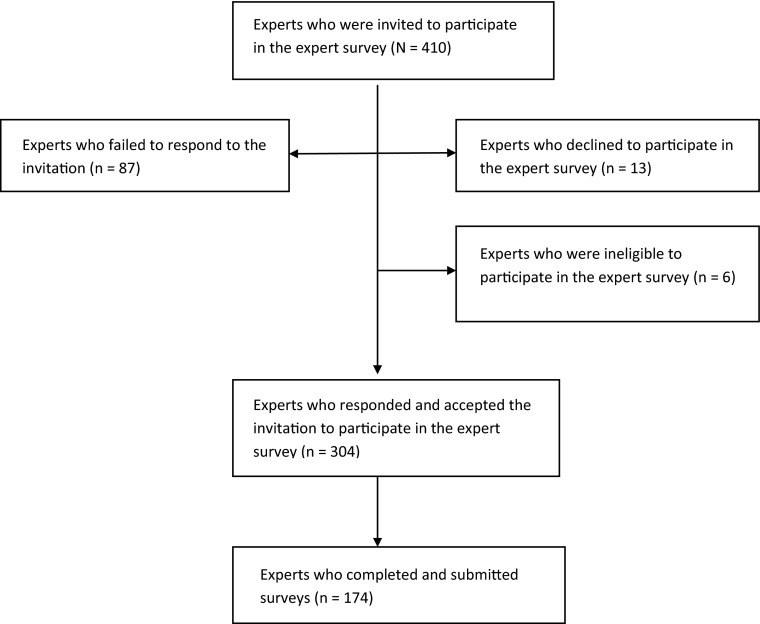
Overview of the expert recruitment process

Invitations were sent to 410 experts. The majority accepted the invitation to participate (*n* = 304; 74 %). Some, however, did not respond to the invitation (*n* = 87), others explicitly declined, mostly (*n* = 9) due to lack of time, but some felt that they were not suited to participate in the expert survey (*n* = 4). A handful of those invited were excluded because they did not meet the inclusion criteria of the study (*n* = 6). Of the 304 experts who initially agreed to participate in the expert survey, 130 failed to submit their survey responses before the data collection window closed. The main reasons given for non-response were lack of time, or “no reason”. An overview of recruitment is provided in Fig. 1.

**Fig. 2:**
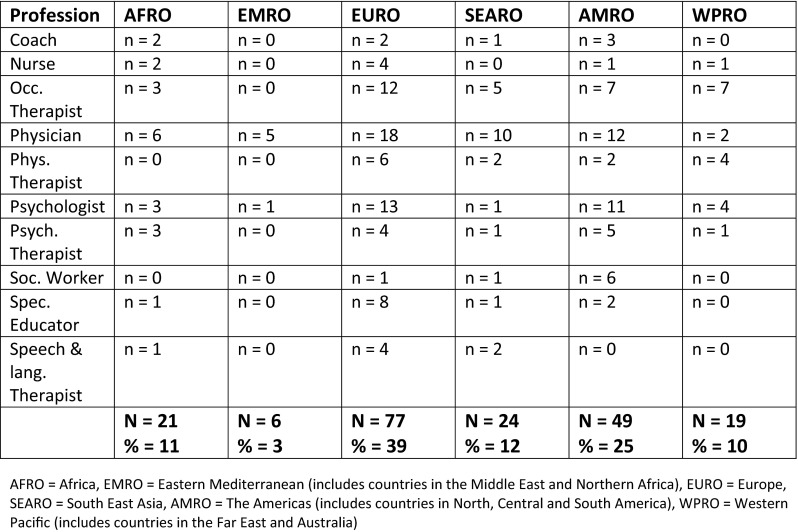
Representation of professions per WHO region (*n* = 196)

Complete survey responses were received from 174 experts (42 %). Some of those that participated declared more than one profession, e.g. psychologist and psychotherapist. Participating experts represented 11 different professional groups, and 45 different countries from all six of the WHO regions. The representation from each of the professions in each of the WHO regions is detailed in Fig. 2. More than half of the participating experts were female (67 %). The mean age was 48 years (SD = 10.2, range 27–69 years). On average, the experts had 16 years of experience (SD = 8.4, range 5–41 years). The majority of the experts worked in clinical fields (52 %), with a smaller proportion working in education (12 %), research (6 %), and management (3 %). The remainder of experts (27 %) divided their time between two or more of these fields, with most combining clinical work with work in other fields. The majority of experts combined the work with children and adolescents (41 %), adolescents and adults (6 %), or worked with clients across the life span (12 %).Thirty percent of participating experts worked exclusively with children, 9 % worked only with adults and 2 % worked only with adolescents.

### Linking results

Analysis of the 174 expert survey responses yielded a total of 7615 meaningful concepts. These concepts were translated and summarised into 185 second-level ICF-CY categories, 235 Personal factors (e.g. self-esteem, sense of humour, resilience), 470 not definable codes (e.g. unstructured environment, self-regulation, social skills), 267 not covered codes (e.g. stress, delinquent behaviour, support programmes for parents), and 55 health condition codes (e.g. depression, conduct disorder, obsessive compulsive disorder). The not definable codes and not covered codes were mostly identified in the questions that captured the environmental factors of functioning and disability, whereas the health condition codes were mostly applied in questions related to Body functions and Body structures component.

Of the identified ICF-CY categories, 83 were found in the responses of at least 5 % of the experts (range: 5–85 %), and were included in the list of candidate categories. Categories were identified in each of the four components making up the ICF, i.e. Body functions (*n* = 30), Activities and Participation (*n* = 30), Environmental factors (*n* = 20) and Body structures (*n* = 3). Table 2 presents the second-level categories that were identified in the Body functions component, along with the number and percentage of expert survey responses in which they were identified. The majority of categories in this component were identified in chapter b1 mental functions (e.g. cognitive, language, emotional functions of the brain; *n* = 19). The other categories were identified in chapters b2 sensory functions and pain (e.g. dizziness, sensation of pain; *n* = 7), b7 neuromusculoskeletal- and movement-related functions (e.g. coordination, clumsiness; *n* = 3), and b3 voice and speech functions (e.g. fluency and rhythm of speech; *n* = 1). Six second-level categories in this component were identified in more than 50 % of the responses. The three most identified categories represent the core symptoms of ADHD, i.e. inattention (b140 attention functions, 85 %), impulsivity (b130 energy and drive functions, 73 %), and hyperactivity (b147 psychomotor functions, 71 %). The three next highest rated categories represent functions related to control and coordination of movement (b760 control of voluntary movement functions, 65 %), a variety of executive functions (b164 higher-level cognitive functions, 61 %), and functions of regulation and display of experienced emotions (b152 emotional functions, 51 %). Most of the second-level categories in Body functions were identified in less than 25 % of the responses (*n* = 21).

**Table 2:** Absolute and relative frequencies of ICF-CY categories from the body functions component

Second-level category	*n* (%)
b140 attention functions	149 (85)
b130 energy and drive functions	127 (73)
b147 psychomotor functions	124 (71)
b760 control of voluntary movement functions	114 (65)
b164 higher-level cognitive functions	106 (61)
b152 emotional functions	90 (51)
b126 temperament and personality functions	64 (37)
b125 dispositions and intra-personal functions	58 (33)
b144 memory functions	55 (31)
b117 intellectual functions	31 (18)
b156 perceptual functions	31 (18)
b134 sleep functions	30 (17)
b180 experience of self and time functions	26 (15)
b235 vestibular functions	19 (11)
b735 muscle tone functions	15 (9)
b260 proprioceptive functions	14 (8)
b160 thought functions	12 (7)
b210 seeing functions	12 (7)
b230 hearing functions	11 (6)
b110 consciousness functions	10 (6)
b122 global psychosocial functions	10 (6)
b163 basic cognitive functions	10 (6)
b114 orientation functions	9 (5)
b167 mental functions of language	9 (5)
b280 sensation of pain	9 (5)
b330 fluency and rhythm of speech functions	9 (5)
b765 involuntary movement functions	9 (5)
b176 mental function of sequencing complex movements	8 (5)
b265 touch function	8 (5)
b270 sensory functions related to temperature and other stimuli	8 (5)

Absolute and relative frequencies of the second-level categories identified in the activities and participation component are presented in Table 3. Identified categories are spread across eight of the nine chapters, i.e. d8 major life areas (e.g. school, work and social life; *n* = 7), d7 interpersonal interactions and relationships (e.g. initiating contact, forming and maintaining specific relationships; *n* = 6), d1 learning and applying knowledge (e.g. reading, writing, focusing attention; *n* = 5), d2 general tasks and demands (e.g. planning and undertaking tasks, managing daily life; *n* = 5), d5 self-care (e.g. washing, eating, toileting; *n* = 3), d4 mobility (e.g. moving around, specific motor skills; *n* = 2), d6 domestic life (e.g. maintaining a household, preparing meals; *n* = 1), and d9 community, social and civic life (e.g. formal and informal socialising, sports; *n* = 1). One second-level category was identified in more than 50 % of the responses, i.e. d820 school education. This category includes various aspects of following and completing an educational programme, such as attending school regularly, cooperating with other students, taking directions from teachers and organising study tasks. As is the case with Body functions, most of the second-level categories in the activities and participation component were identified in less than 25 % of the responses (*n* = 22).

**Table 3:** Absolute and relative frequencies of ICF-CY categories from the activities and participation component

Second-level category	*n* (%)
d820 school education	91 (52)
d570 looking after one’s health	68 (39)
d250 managing one’s own behaviour	67 (38)
d750 informal social relationships	67 (38)
d230 carrying out daily routine	60 (34)
d720 complex interpersonal interactions	60 (34)
d440 fine hand use	59 (34)
d210 undertaking a single task	49 (28)
d161 directing attention	41 (23)
d760 family relationships	41 (23)
d446 fine foot use	38 (22)
d920 recreation and leisure	35 (20)
d240 handling stress and other psychological demands	32 (18)
d220 undertaking multiple tasks	31 (18)
d830 higher education	26 (15)
d850 remunerative employment	26 (15)
d571 looking after one’s safety	25 (14)
d710 basic interpersonal interactions	22 (13)
d825 vocational training	22 (13)
d845 acquiring, keeping and terminating a job	21 (12)
d740 formal relationships	20 (11)
d170 writing	15 (9)
d870 economic self-sufficiency	15 (9)
d880 engagement in play	14 (8)
d115 listening	13 (7)
d160 focusing attention	13 (7)
d770 intimate relationships	13 (7)
d166 reading	10 (6)
d540 dressing	8 (5)
d640 doing housework	8 (5)

Table 4 shows the absolute and relative frequencies of the second-level categories that were identified in the Environmental factors component. Categories in this component were identified in all five chapters, i.e. e3 support and relationships (e.g. support from family, friends and colleagues; *n* = 7), e1 products and technology (e.g. products for use in daily living, education, communication; *n* = 5), e4 attitudes (e.g. attitudes of friends, health professionals, societal attitudes that influence individual behaviour and social life; *n* = 4), e5 services, systems and policies (e.g. health services, special education; *n* = 3), and e2 natural environment and human-made changes to environment (e.g. light, sound, climate; *n* = 1). Two of the 20 second-level categories identified in this component were found in more than 50 % of the responses. The first is e310 immediate family, representing the support provided by parents, partners, siblings and other direct family members. The second is e585 education and training services, systems and policies, involving institutions providing mainstream and special education and related services. Environmental factors indicated by the experts can be either helpful or hindering, such as e110 products or substances for personal consumption, which can be a facilitator in the form of medication, but a barrier when it represents alcohol or other non-medical drugs related to substance abuse. The majority of the second-level categories in this component were again identified in less than 25 % of the responses (*n* = 16).

**Table 4:** Absolute and relative frequencies of ICF-CY categories from the *Environmental factors* component

Second-level category	N (%)
e310 immediate family	107 (61)
e585 education and training services, systems and policies	94 (54)
e580 health services, systems and policies	51 (29)
e410 individual attitudes of immediate family members	45 (26)
e115 products and technology for personal use in daily living	38 (22)
e360 other professionals	37 (21)
e455 individual attitudes of other professionals	37 (21)
e110 products or substances for personal consumption	25 (14)
e425 individual attitudes of acquaintances, peers, colleagues, neighbours and community members	17 (10)
e340 personal care providers and personal assistants	16 (9)
e460 societal attitudes	16 (9)
e250 sound	15 (9)
e325 acquaintances, peers, colleagues, neighbours and community members	14 (8)
e320 friends	12 (7)
e165 assets	11 (6)
e355 health professionals	10 (6)
e590 labour and employment services, systems and policies	10 (6)
e125 products and technology for communication	9 (5)
e130 products and technology for education	9 (5)
e330 people in positions of authority	8 (5)

The three second-level categories identified in the body structures component are shown in Table 5, along with their absolute and relative frequencies. One of these was identified in 83 % of the responses, i.e. s110 structure of brain from chapter s1 structures of the nervous system. This category represents abnormalities that may occur in the various structures of the brain, such as the brain stem, the different cortical lobes and the amygdala. The other two categories were identified in chapter s7 structures related to movement. The first, s730 structure of upper extremity, represents problems that may occur in the arms, hands and fingers, whereas the second, s750 structure of lower extremity, may indicate problems in the legs, ankles and feet. Both of these categories were identified in fewer than 25 % of the responses.

**Table 5:** Absolute and relative frequencies of ICF-CY categories from the Body structures component

Second-level category	*n* (%)
s110 structure of brain	146 (83)
s730 structure of upper extremity	22 (13)
s750 structure of lower extremity	19 (11)

Additional frequency analysis of the expert survey responses exploring the possible relationship between age groups (children, adolescents, adults) and the ICF-CY categories identified in their survey responses showed that the majority of categories identified by specialists were from across the lifespan. A few categories were identified more often (*n* = 34) by those working with adults as compared to those working with children: d845 acquiring, keeping and terminating a job, d850 remunerative employment and d870 economic self-sufficiency, as were categories d230 carrying out daily routine, and d570 looking after one’s health. In the activities covered by these categories, children are often guided by adults, rather than being responsible for themselves. Two additional categories were identified by experts working with adults, but less frequently than those working with children, i.e. b125 dispositions and intra-personal functions (such as adaptability and energy level) and b134 sleep functions (including onset, maintenance and quality of sleep). Finally, one category was identified more often (n = 71) by those working with ADHD children compared to adults, namely e585 education and training services, systems and policies.

### ADHD-related abilities

The vast majority of experts (93 %) indicated positive sides to ADHD and named one or more abilities related to the condition. There were some recurring themes. A high level of energy was often mentioned as a strength making individuals with ADHD lively and dynamic, often exciting and fun to have around, and enabling them sometimes to get a lot of things done. Other positive characteristics often mentioned were flexibility, resiliency, perseverance, creativity and a generally optimistic attitude, as well as the ability to multitask and process input from various sources simultaneously. Experts described individuals with ADHD as passionate and having a strong drive for the things that interest and motivate them. This was said to make them persist and work hard on these things with a focus that can exceed that of others, and enables them to inspire and energise those around them. Furthermore, they were described as fast learners, fast thinkers and fast decision makers, unafraid to take risks, and not easily discouraged by obstacles. Towards others they were generally described as being sociable, caring, sensitive to the moods and feelings of others as well as loyal, noble and altruistic. It was generally felt that a requirement for these strengths and abilities to become apparent in ADHD is that individuals receive appropriate support or treatment for their needs. An overview of the abilities and strengths mentioned by the experts can be found in Table 6.

**Table 6:** Overview of recurrent abilities and strengths mentioned by the experts

Abilities and skills
Creativity
Energetic
Exciting and fun to be around
Flexibility
Interesting view on things
Multitasking
Resilience
Risk takers

### Gender differences

A majority of experts (65 %) identified gender differences. Of these more than ¾ reported that they had experienced these differences in their daily practice. The most frequently described difference was that males more often presented with the hyperactive/impulsive subtype and females more often presented with the inattentive subtype of ADHD. More specifically, males appear to present with more externalising behaviour problems, more bullying and aggressive behaviours, more social problems and problems with authority, and a greater tendency towards addictive behaviours and substance abuse. Females were reported to have more internalising problems like anxiety and depression, more often feel stressed and overwhelmed trying to live up to social expectations, have lower self-esteem, and more often show self-injurious behaviour and suicidal attempts. Females were also reported to make greater efforts, and to more succeed to hide their difficulties than males. As a consequence, males with ADHD were reported to be generally more readily recognised and diagnosed, in contrast to females whose symptoms and problems often remain unnoticed and where diagnosis occurs often later in life.

## Discussion

The current study aimed to capture an expert perspective on the abilities and disabilities related to ADHD through a worldwide survey of ADHD experts from different professional and cultural backgrounds. The experiences and opinions of such a large and diverse group of ADHD experts have not previously been documented in this extensive way. Therefore, the results provide global and diverse insights into ability and disability related to ADHD.

With a large number of ICF-CY categories identified across all of the components, the results support the notion that ADHD impacts on major areas of everyday life. From the Body functions component, nearly two-thirds of the identified categories are from chapter b1 mental functions. This finding is perhaps not surprising as ADHD is a mental health condition and thus is expected to affect primarily mental functions. Likewise, the only category in the Body structure component named by the majority of the experts was s110 structure of brain. However, the fact that no fewer than 19 different mental functions were indicated by at least 5 % of the experts shows that ADHD is experienced to affect a far broader composition of mental functions than the core behavioural domains of inattention (b140), impulsivity (b130) and hyperactivity (b147). Some physical functions were also included in the expert responses, suggesting that there are also physical challenges associated with ADHD. These concerned mostly sensory processing and motor-related functions. In the activities and participation component, all chapters except (d3) communication were represented in the selection. This suggests that activities and participation in all areas of life, ranging from managing oneself and everyday demands to relationships with others, education, employment, and recreation, are affected in ADHD. The same can be said about environmental factors, which are represented by categories from all of its chapters. It is important to note that environmental factors can represent both factors that are hindering (i.e. barriers) or helpful (i.e. facilitators) for the individual. For example, (e585) education and training services, systems and policies could be named by an expert as a facilitator when the school provides adequate support for the specific needs of the student with ADHD. However, the same category might represent a barrier when the school does not acknowledge ADHD or a child’s behavioural problems as a health condition. The same goes for the other categories identified in the Environmental factors component.

This study is amongst the first to explore specific strengths and gender differences in ADHD as perceived by experts. Overall, the opinions proposed were too broad and inconsistent to derive meaningful concepts and link ICF categories. Thus, this aspect of the survey data was analysed in an exploratory way. Many experts reported both certain abilities linked to ADHD and phenotypes related to gender in ADHD. Whilst there is accumulating evidence indicating subtle, but important gender differences in ADHD [33], there is little empirical support for certain abilities and skills related to ADHD that have been mentioned by the experts in the survey, such as enhanced creativity or fast processing speed. [34]. Therefore, in part these multidisciplinary perspectives may reflect personal anecdotes that are influenced by one’s professional training and working experience. However, the expert survey data on these topics demonstrated that ADHD experts do not only identify deficits, but also have a positive outlook on the impact of ADHD, and a widespread awareness that gender issues may be important for fully understanding ADHD. Furthermore, they generate novel hypotheses that can be tested in future studies. Examples include increased optimism and hyper-performance under certain circumstances or increased efforts by females with ADHD to hide other difficulties. This study did not investigate whether experienced strengths or gender differences were related to WHO-regional differences, but we hope to report such possible cultural differences in a later paper combining the expert survey data with findings from the upcoming preparatory focus groups and clinical study.

The survey did present some challenges. For example, there were many meaningful concepts in the expert responses for which no appropriate ICF-CY category could be identified. This resulted in a large number of not definable codes (*n* = 470). This can be explained in part by the fact that experts answered the survey questions using their own words and formulations. For the linking, this often presented a challenge since answers were sometimes very brief or ambiguous and, therefore, difficult to properly interpret and link (e.g. single word answers such as “familiarity” or “gadgets”). These types of answers were most commonly responses to the questions related to supportive and hindering Environmental factors. In other cases, answers were formulated in such general terms that no one specific ICF-CY category could be identified to represent them (e.g. “supportive environment” or “behaviour problems”). These were again often in response to the questions that captured the Environmental factors of functioning and impairment. However, a closer look at the not definable codes did reveal a few recurring themes. For example, a large proportion of the codes represent the level of structure and routine present in the environment.

One barrier to functioning indicated by the experts was a lack of structure (e.g. “chaotic environment”, “poor structure”), whilst higher levels of structure were considered facilitators for individuals with ADHD (e.g. “clear structure” and “daily routine”).Another frequently recurring theme was distractions and overstimulation in the environment. These were named by experts as significant barriers to the functioning of individuals with ADHD (e.g. “cognitive overload”, “emotional overload”, and “noisy work environment”). Finally, many of the not definable codes represented social and behavioural problems, such as “social awkwardness” and “behavioural control”.

Another challenge in the interpretation of findings is the fact that categories in the Environmental factors component in the ICF(-CY) are formulated in a very general way. For example, the chapter (e3) support and relationships include categories that describe only the people or relationships that may represent facilitators or barriers for the individual, such as (e310) immediate family and (e355) health professionals. Unfortunately, the categories do not include any indication of how (relationships with) these people can be helpful or hindering to the individual. The same is true for (e115) products and technology for personal use in daily living, which includes no further description of these products and technologies or how they may be helpful or hindering to the person. This makes it difficult to draw any conclusions extending beyond saying that certain products and technologies used in daily life are helpful for individuals with ADHD. More detailed descriptions and further specifications of categories in the Environmental factor component in the ICF(-CY) would be helpful to create a more complete picture of how an individual’s level of functioning is shaped in relation to the environment. Challenges like these remind us that the current first edition of the ICF(-CY) originates rather from the field of physical than mental health and there is the potential for considerable improvement in relation to the assessment of functioning in the context of mental health problems.

Whilst the current study provides a unique, global, multidisciplinary expert perspective on abilities and disabilities associated with ADHD, it cannot be assumed that it is complete, exhaustive or representative for all experts around the world. Even though experts included in the study represented a broad range of different professions and all six of the WHO regions, some professions had only limited representation and some were not represented in all regions. Moreover, the response rate of experts who were invited to participate in this study was modest. Therefore, it seems likely that the identified categories reflect at least in part the composition of those experts who were included. In part the composition of the sample reflects the fact that certain professions either do not exist in some parts of the world, or are not involved in the assessment and treatment of ADHD. For example nurses are not involved in ADHD treatment in India (SEARO region). Similarly, the diagnosis of ADHD is not yet known or not generally accepted or prioritised in some countries within certain regions, such as the Middle East (e.g. Afghanistan) and Africa (e.g. Ghana).The requirement for experts to be able to communicate in English will also have limited those who were able to take part in the study. Several experts were identified and invited to participate in the study, but declined due to not having sufficient English language skills required for completing the survey. This was mostly commonly an issue in regions where English is not commonly used in communication, e.g. South American countries in the AMRO region and Far-Eastern countries in the WPRO region. Specific efforts were, however, made to include at least some experts from these countries and regions in this study. The fact that an email survey was used to survey the experts represented a limitation in itself, both in terms of global reach (many low- and middle-income countries do not have easy access to internet), and because it was not possible to know whether experts interpreted the questions as intended. Extreme care was taken to formulate the survey questions in such a way as to facilitate participation of a broad range of participants. Finally, a technical limitation to the study resulted in 4 experts being unable to provide their responses to the survey due to problems opening and working with the files sent to them via email. However, due to the small number of participants being affected by these problems this is unlikely to have affected the study validity.

It is important to see the findings of the current study within the context of the larger project. This is the second study in a total of four that will together inform the development of the ICF Core Sets for ADHD. The first, the comprehensive scoping literature review, aimed to capture the research perspective of ability and disability related to ADHD, and was published earlier [35]. The current study identified that ADHD is not only related to disability, but also to strengths and abilities (e.g. creativity, high level of energy and flexibility). This contrasts with the results of the comprehensive scoping review, which identified disabilities but not strengths. Moreover, categories from the Body structure component (e.g. brain and extremities) were identified in this study, but not in the literature review. In addition, the comprehensive scoping review yielded only a limited number of categories from the Environmental factors component, whereas the current study identified many ICF-CY categories related to environmental issues. Consistent with the scoping literature review, the three most frequently identified categories in the Body functions component in this study also represented the core domains of ADHD, namely attention (b140), impulse control (b130), and hyperactivity (b147). The two remaining studies are a qualitative focus group study, which will capture the perspectives of individuals with ADHD and their families, and a cross-sectional clinical study, which will capture abilities and disabilities of actual patients in real-life settings. Together these four studies are designed to identify and describe the full spectrum of function and dysfunction that are specifically relevant to ADHD and which will form the scientific basis for the formulation of ICF Core Sets for ADHD. The process was designed by the WHO and the ICF Research Branch, a partner of the WHO Collaboration Centre for the Family of International Classifications in Germany (at DIMDI), to ensure that a global perspective on functioning in a certain health condition is captured and that the ICF Core Sets are universally applicable. Including the four complementary preparatory studies in this process makes it possible for one study to fill in the gaps that were the result of the limitations of another study. For example, certain countries or regions may be underrepresented in this study and even in the scoping literature review (given the requirement for publications to be in English), but these will be included in the upcoming focus group and clinical studies. The language should not be a barrier in these studies, since collaborations with members from the Steering Committee and other international study sites will allow for the use of local languages. In another example, focus group discussions with individuals with ADHD and their close relations will make it possible to capture specific aspects of functioning in ADHD that may be overlooked by researchers or clinicians, since they are experienced primarily by individuals living with ADHD. Together the results of the four studies should provide a comprehensive picture of the specific abilities and disabilities that are related to functioning in ADHD for individuals across different countries, cultures and life situations. In turn, these results combined will provide the input for an international consensus conference during which a group of ADHD experts from all WHO regions will discuss and decide which of the ICF-CY categories should be included in the ICF Core Sets for ADHD. The final result of these efforts will be ICF Core Sets that provide a globally representative and universally applicable standard for the assessment of functioning in individuals with ADHD, equally useful in both clinical practice and scientific research.
